# Acute prefrontal hemodynamic responses to Xingnao Kaiqiao acupuncture in healthy participants and patients with prolonged disorders of consciousness

**DOI:** 10.3389/fneur.2026.1889514

**Published:** 2026-08-28

**Authors:** Haibo Guo, Linlin Liu, Zihao Sun, Hongzhen Du, Yuxuan Zhang, Shutong Mei, Yang Wang, Zhijie Cao

**Affiliations:** 1 Department of Rehabilitation Medicine, Binzhou Medical University Hospital, Binzhou, Shandong, China; 2 School of Special Education and Rehabilitation, Shandong Medical and Pharmaceutical University, Yantai, Shandong, China

**Keywords:** functional connectivity, functional near-infrared spectroscopy, prolonged disorders of consciousness, sham control, Xingnao Kaiqiao acupuncture

## Abstract

**Background/objectives:**

Xingnao Kaiqiao (XNKQ) acupuncture is used clinically in prolonged disorders of consciousness (pDOC), but the cortical processes underlying its acute effects remain incompletely characterised. We used bedside functional near-infrared spectroscopy (fNIRS) with a superficial non-acupoint sham comparator to examine acute prefrontal hemodynamic responses in pDOC patients and healthy controls.

**Methods:**

Forty-five pDOC patients (25 minimally conscious state; 20 unresponsive wakefulness syndrome) and 127 healthy volunteers were enrolled; 124 healthy volunteers were retained for complete-case analysis. Participants received real XNKQ acupuncture or superficial sham needling at adjacent non-acupoints. fNIRS recorded oxyhaemoglobin (HbO) across three 8-min windows: pre-acupuncture (Before), steady-state needle retention (During), and post-acupuncture (After). Regional HbO was the principal analysis; connectivity-derived network, temporal, and baseline-association analyses were exploratory.

**Results:**

In healthy participants, real acupuncture produced greater HbO responses than sham across all four regions and the global prefrontal average (Cohen’s *d* = 0.62–1.20; all *q* ≤ 0.0011). In pDOC patients, the response was smaller and regionally restricted: the dorsolateral prefrontal cortex (DLPFC; *d* = 0.80, *q* = 0.0397) and global HbO (*d* = 0.98, *q* = 0.0191) remained significant after false discovery rate (FDR) correction, whereas the frontopolar area (PFA) was borderline (*q* = 0.0576), and the sensorimotor cortex and inferior frontal area (IFA) were not significant. Baseline-adjusted analyses showed the same general pattern. No connectivity-derived network contrast survived FDR correction. Baseline-to-change correlations were negative but mathematically coupled.

**Conclusion:**

Real XNKQ acupuncture was associated with greater acute prefrontal HbO responses than superficial non-acupoint needling. The effect was broad in healthy participants and more restricted in pDOC patients. These findings demonstrate an acute hemodynamic response, not clinical efficacy, preserved awareness, or strict acupoint specificity. Network and baseline association findings remain exploratory.

**Patient protocol registration:**

Chinese Clinical Trial Registry, https://www.chictr.org.cn, identifier ChiCTR2400090915 (registered 15 October 2024).

## 1 Introduction

Prolonged disorders of consciousness (pDOC), which comprise the unresponsive wakefulness syndrome (UWS) and the minimally conscious state (MCS), are among the most challenging outcomes of severe acquired brain injury and frequently lead to long-term severe disability. With continuing advances in critical care and resuscitation, the number of patients surviving with a prolonged disorder of consciousness is increasing worldwide; the reported prevalence of UWS ranges from 0.2 to 6.1 per 100,000 in European surveys, and an estimated 100,000 to 300,000 patients are living with a prolonged disorder of consciousness in the United States (1, 2). Treatment options remain limited. Amantadine is the only pharmacological agent with Level I evidence (3). A network meta-analysis of 32 randomised controlled trials (1,770 patients) found that several non-pharmacological interventions, including repetitive transcranial magnetic stimulation, transcranial direct-current stimulation, median-nerve electrical stimulation, hyperbaric oxygen, and acupuncture, all significantly improved Coma Recovery Scale-Revised (CRS-R) scores (4). Among these interventions, acupuncture is one of the most widely used in China; the cortical mechanisms underlying its effect, however, remain incompletely understood, which limits protocol optimisation and broader clinical adoption.

Xingnao Kaiqiao (XNKQ) acupuncture is a semi-standardised manual acupuncture technique developed by Xuemin Shi for neurological conditions, particularly stroke (5, 6). The core prescription comprises Renzhong (DU26), bilateral Neiguan (PC6), and bilateral Sanyinjiao (SP6), with Jiquan (HT1), Chize (LU5), and Weizhong (BL40) added to the clinical routine (6). The protocol provides somatosensory input from facial and upper- and lower-limb sites. Neuroimaging and near-infrared spectroscopy studies at PC6, DU26, and SP6 have reported changes in distributed brain activity or cerebral oxygenation (7–9). These observations support multisite somatosensory engagement, but they do not establish a unique afferent pathway or an acupoint-specific mechanism for any single point. The clinical evidence for XNKQ in pDOC is summarised in recent meta-analyses (4), but acute cortical mechanisms remain insufficiently defined.

The choice of imaging modality is a practical constraint for studying acupuncture in this population. Functional magnetic resonance imaging is incompatible with metal needles and requires patients to remain still in a confined scanner. Electroencephalography offers high temporal resolution but only limited spatial localisation of cortical sources. Functional near-infrared spectroscopy (fNIRS) circumvents both limitations: it is silent, portable, tolerant of mild movement, and fully compatible with metal needles, allowing continuous bedside recording throughout the acupuncture procedure (10). Recent reviews of fNIRS studies in traditional Chinese medicine non-pharmacological interventions support the feasibility of fNIRS for characterising cortical responses across different acupuncture conditions, manipulation parameters, and deqi-related states (11, 12). A recent sham-controlled study further supports its use in capturing acute prefrontal responses during acupuncture (13). Connectivity-based measures are closely linked to the level of consciousness: the recovery of cortical effective connectivity accompanies the recovery of consciousness in patients emerging from the vegetative state (14). Resting-state fNIRS studies have shown that pDOC patients exhibit disrupted prefrontal functional connectivity and altered graph-theoretic network topology (15–17), and that network features can discriminate consciousness levels with clinically meaningful accuracy (17).

Si et al. (18) demonstrated in healthy participants that fNIRS can detect differential prefrontal and motor cortical responses between manual acupuncture with deqi and tactile control stimulation. In pDOC, Wen et al. (8) recorded increased prefrontal HbO during acupuncture in 16 patients without a sham comparator, and Liu et al. (19) randomly assigned 28 pDOC patients to real or sham acupuncture, reporting dorsolateral prefrontal cortex (DLPFC), primary motor cortex (M1), and primary somatosensory cortex (S1) activation and functional connectivity changes after one session. These studies established the feasibility of bedside fNIRS during acupuncture, but three issues remain: first, no study has included a healthy comparison cohort; second, previous studies have focused on activation or pairwise connectivity rather than network-level topology; and third, no study has examined how baseline network state relates to within-session change. Recent fNIRS studies from our institution (20) confirmed bedside fNIRS feasibility in pDOC patients (emotional-auditory paradigm) but did not examine acupuncture-related responses. Recent studies have further established fNIRS as a bedside tool for detecting residual cortical processing, command-following, and covert awareness after acute severe brain injury (21–23).

Here, we combined 35-channel fNIRS with a sham-controlled design in 124 healthy volunteers and 45 pDOC patients to: (1) determine whether real XNKQ acupuncture produces greater acute prefrontal HbO responses than superficial non-acupoint needling; (2) test whether the magnitude of this contrast differs between healthy and pDOC cohorts; and (3) describe exploratory connectivity-derived network, temporal, and baseline-association findings. We hypothesised that the real-versus-sham HbO contrast would be larger in healthy participants and more regionally restricted in pDOC patients.

## 2 Materials and methods

### 2.1 Study design and participants

The patient component was a prospective, randomised, sham-controlled study conducted at the Department of Rehabilitation Medicine, Binzhou Medical University Hospital, between October 2024 and June 2025. The patient acupuncture protocol was registered on the Chinese Clinical Trial Registry (ChiCTR2400090915; registered 15 October 2024). The healthy-participant cohort was included as a parallel physiological comparator and was not part of the registered patient trial. It served as an age-comparable physiological reference rather than a formally age- and sex-matched clinical control group; direct cross-cohort comparisons were therefore interpreted cautiously. The protocol was approved by the hospital ethics committee (KYLL-186). Written informed consent was obtained directly from all healthy volunteers and from the legally authorised representatives of all pDOC patients.

We enrolled 45 pDOC patients (mean age 55.33 ± 16.18 years, 23 female) and 127 healthy volunteers (mean age 56.33 ± 10.26 years, 25 female); three healthy participants with incomplete three-phase fNIRS data were excluded, leaving 124 healthy volunteers for complete-case analysis. Of the 45 patients, 25 were classified as MCS and 20 as UWS based on the Coma Recovery Scale-Revised (CRS-R) using the standard diagnostic criteria (24), which classifies a participant as UWS when no MCS-defining behaviours are observed across the six CRS-R subscales, and as MCS when at least one such behaviour is consistently present. Patient inclusion criteria were: age 18–80 years; diagnosis of pDOC (>28 days post-injury) classified as UWS or MCS by CRS-R; etiology of stroke, hypoxic–ischaemic encephalopathy, or traumatic brain injury; and stable vital signs. Exclusion criteria were: skull defects, craniotomy, or hydrocephalus affecting fNIRS signal acquisition; uncontrolled epilepsy; sedative medication; severe cardiopulmonary, hepatic, or renal dysfunction; scalp injury or infection; and history of psychiatric or neurodegenerative disease. The CRS-R was administered on five non-consecutive days within a 10-day window by the same trained physician.

**Figure 1:**
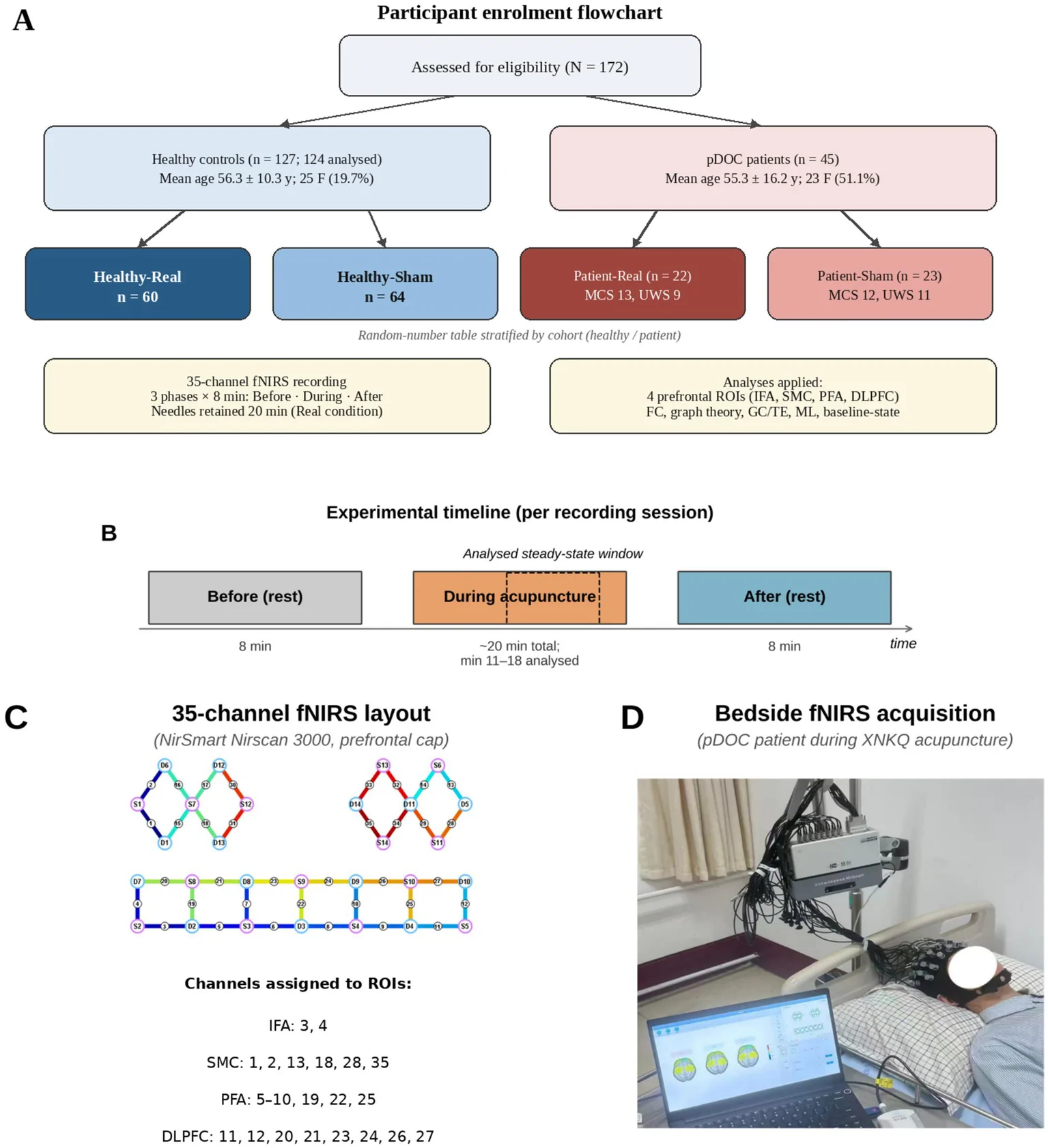
Study design and fNIRS setup. **(A)** CONSORT-style flow shows enrollment of 127 healthy volunteers and 45 pDOC patients, with 124 healthy volunteers and all 45 patients included in the complete-case analysis. **(B)** Experimental timeline of the three 8-min analysis windows (Before/During steady-state retention/After). **(C)** 35-channel fNIRS layout colour-coded by ROI: IFA (red), SMC (orange), PFA (green), DLPFC (blue). **(D)** Bedside acquisition photograph showing continuous fNIRS recording during XNKQ acupuncture with sterile Huatuo stainless-steel needles (0.30 × 40 mm).

Allocation to the real or sham subgroup was determined by a computer-generated random-number table prepared before enrolment, with stratification by cohort (healthy/patient). Twenty-two patients were assigned to real XNKQ (13 MCS, 9 UWS) and 23 to sham (12 MCS, 11 UWS); 63 healthy volunteers received real and 64 sham; after exclusion, 60 healthy participants in the real subgroup and 64 in the sham subgroup contributed to the analysed dataset. The four analysis groups are Healthy–Real (*n* = 60), Healthy–Sham (*n* = 64), Patient–Real (*n* = 22), and Patient–Sham (*n* = 23). Baseline characteristics did not differ between real and sham subgroups within each cohort (all *p* > 0.05). The CONSORT-style flow is shown in Figure 1A.

Sample size was estimated *a priori* using G*Power 3.1.9.7. For an independent-sample *t*-test (two-tailed), assuming a large effect (Cohen’s *d* = 0.85), with *α* = 0.05, power (1 − *β*) = 0.80, and 1:1 allocation, a minimum of 23 participants per group was required. The healthy cohort substantially exceeded this requirement, and the patient subgroups (real acupuncture, *n* = 22; sham, *n* = 23) were at or close to the estimated minimum; the primary Healthy–Real versus Healthy–Sham comparison was powered above 0.95.

### 2.2 Instrumentation and recording

Cortical hemodynamics were recorded with a 35-channel continuous-wave fNIRS system (NirSmart Nirscan 3000, Huichuang Medical, Danyang, China) at 11 Hz (730/850 nm). Sixteen sources and 16 detectors cover the bilateral prefrontal cortex, with source–detector separation measures 2.9–3.1 cm. The cap was positioned according to the international 10–20 system, using Fpz and Cz as positioning landmarks, with Fpz aligned to the nasion. During acquisition, the manufacturer’s real-time coefficient-of-variation criterion (<15%) was used to optimise optode contact; this online acquisition cheque was distinct from the offline SCI-based channel pruning described below. Channels were assigned to four regions of interest (ROIs) by Brodmann mapping (Figure 1C): inferior frontal area (IFA, channels 3–4, Brodmann areas (BAs) 44/45); sensorimotor cortex (SMC, channels 1, 2, 13, 18, 28, 35; BAs 4/6); frontopolar area (PFA, channels 5–10, 19, 22, 25; BA 10); and dorsolateral prefrontal cortex (DLPFC, channels 11, 12, 20, 21, 23, 24, 26, 27; BA 9/46).

Each session comprises three consecutive phases (Figure 1B). Before: 8-min pre-acupuncture resting-state recording. During: continuous fNIRS through the ~20-min acupuncture procedure. After: 8-min resting-state recording started immediately after needle removal. For analysis, three 8-min segments were extracted: the Before window; the steady-state retention window (minutes 11 to 18 of the During phase, after manipulation was complete and the needles were retained without further intervention; this window was applied identically to all participants, with no protocol deviations); and the After window. This avoids contaminating the analysed window with the brief manipulation period at the start of needle retention.

### 2.3 Acupuncture procedure

Real acupuncture uses sterile single-use stainless-steel needles (0.30 × 40 mm; Huatuo brand, Suzhou Medical Appliance Factory, Suzhou, China). PC6: perpendicular insertion 0.5–1.0 cun with combined lifting–thrusting and twirling, reducing manipulation at 120–160·min^−^1 for 1 min. DU26: oblique insertion toward the nasal septum 0.3–0.5 cun, with pecking manipulation at 180–200·min^−^1 until ocular moistening. SP6: oblique insertion along the medial tibial border 0.5–1.0 cun with reinforcing manipulation (gentle twirling, slow insertion, heavy pressing, slow lifting) for 1 min. HT1, LU5, BL40: perpendicular 0.5–1.0 cun, reducing manipulation until a limb twitch was observed (three times). Needles were retained for 20 min without further manipulation. Sham acupuncture used identical needles inserted superficially (2–3 mm, not exceeding subcutaneous tissue) at non-acupoint locations 2.5 cm lateral to each real acupoint, without any manipulation. All procedures were performed by a single licensed acupuncturist with more than 10 years of clinical experience and XNKQ certification. fNIRS data were analysed by a researcher blinded to group allocation. The acupuncturist delivering the intervention could not be blinded, as is inherent to acupuncture trials, and formal blinding of participants and clinical staff was not implemented or tested; because expectancy and practitioner behaviour can influence acupuncture responses, this is acknowledged as a limitation and should be addressed in future studies. Acupoints and sham points were localised according to the XNKQ protocol, and the intervention is described in accordance with the Standards for Reporting Interventions in Clinical Trials of Acupuncture (STRICTA) 2010 reporting domains, including acupuncture rationale, needling details, treatment regimen, practitioner background, and comparator intervention (25). Subjective deqi ratings were not collected as an outcome because most pDOC patients could not provide reliable sensation reports; this is considered in the interpretation of the real-versus-sham contrast.

### 2.4 Data processing

Recordings were exported from the NirSpark software of the NirSmart system as raw optical-intensity data in Homer (.nirs) format, and optical density was computed as OD = −log₁₀(I/I₀). All preprocessing and analysis steps described below were implemented in Python and produced the results reported here. Motion artefacts were identified and corrected on the two-wavelength optical-density signals before hemodynamic filtering using a moving standard-deviation criterion, spline interpolation, and temporal derivative distribution repair (26–28). After motion correction and before conversion to chromophore concentrations, the scalp coupling index (SCI) was computed from the corrected two-wavelength optical-density signals after isolation of the cardiac band (29). Channels with SCI < 0.75 were excluded from subsequent analyses. Recording-level signal-quality summaries are reported in Section 3.1 and Supplementary Table S12. Qualified optical-density signals were converted to HbO and deoxyhaemoglobin (HbR) concentration changes using the modified Beer–Lambert law with Prahl/Gratzer extinction coefficients at 730 and 850 nm and age-adjusted differential pathlength factors, and then filtered with a zero-phase third-order Butterworth band-pass filter (0.01–0.15 Hz). ROI values were averaged across the retained channels within each ROI. The principal HbO outcomes analysed in this report are the four ROI-averaged HbO values (the regional outcomes) and a global HbO value obtained by averaging across all retained prefrontal channels (the global outcome). This principal regional and global HbO dataset contained no missing outcome values, and no across-participant imputation was used. Analyses focused on HbO because of its higher signal-to-noise ratio; region-level HbR results are reported in Supplementary Table S10. Concentration changes are expressed relative to the mean of the first 20 s of each recording window, with an increase in either chromophore expressed as a positive value, so that the canonical cortical activation pattern would appear as an HbO increase accompanied by an HbR decrease. In the present data, HbR did not show the canonical decrease but changed in the same direction as HbO under real acupuncture in the healthy cohort and in most patient regions, indicating a response dominated by blood-volume change; the region-level HbR results (Supplementary Table S10) are therefore reported descriptively only. Because no short-separation channels were available, extracerebral and systemic contributions cannot be excluded.

Functional connectivity (FC) was computed as Fisher-z-transformed Pearson correlations between channel pairs. Channels that failed the SCI criterion were removed from the connectivity analysis, so that functional connectivity and the graph summaries were computed on the retained-channel subgraph for each participant; connections involving an excluded channel were excluded from the analysis rather than assigned a fixed value or imputed. Region- and global-average time courses were computed by averaging only the retained channels. Because the set of retained channels varied across participants, the number and composition of network nodes differed between individuals, so network summaries were examined on density-matched graphs and are reported as descriptive and exploratory. Pearson’s correlation was retained for comparability with previous fNIRS connectivity studies (30). Because fNIRS signals are non-stationary and may share a global physiological component that inflates inter-channel correlations, signals were restricted to the 0.01–0.15 Hz band and a global component was regressed out before connectivity estimation. Graph summaries followed standard definitions (31, 32) and are illustrated conceptually in Supplementary Figure S2. Density-matched network representations were examined descriptively to reduce dependence on overall connection strength; however, threshold robustness was not treated as established, and no inferential conclusion relies on a particular graph threshold. FC-derived network summaries (degree-like connectivity strength, clustering coefficient, characteristic path length, global efficiency, local efficiency, and small-world index) were therefore retained only as exploratory descriptors of network organisation. Granger causality and transfer entropy were computed at the ROI level, and temporal-dynamic features (peak time, recovery ratio, and slope) were extracted for each ROI.

A secondary machine-learning analysis was retained only as an exploratory internal-validation sensitivity analysis. Random forest, linear support vector machine, and logistic regression were selected to represent complementary non-linear, margin-based linear, and interpretable linear model families. Five-fold cross-validation was grouped by an anonymised participant identifier so that all Before, During, and After recordings from the same participant remained in the same fold. Imputation, standardisation when required, and univariate selection of the top 50 features were fitted exclusively within each training fold and then applied to held-out data. Detailed results are reported only in Supplementary Table S6; no external test set was available, and these analyses are not used to support clinical or diagnostic utility.

### 2.5 Statistical analysis

Continuous variables are reported as the mean ± standard deviation (SD). Normality was assessed by Shapiro–Wilk tests. Baseline and demographic comparisons used independent-sample *t*-tests or Mann–Whitney *U*-tests for continuous variables and *χ*^2^ or Fisher’s exact tests for categorical variables. During-phase real-versus-sham comparisons used Welch independent-samples *t*-tests, with Cohen’s d and bootstrap 95% confidence intervals (2,000 resamples). As robustness analyses, During HbO was additionally modelled by analysis of covariance (ANCOVA) with the corresponding Before value as a covariate, and cohort × treatment interactions were estimated with heteroscedasticity-robust linear models. Multiple-comparison correction was used with Benjamini–Hochberg false discovery rate (FDR) within each cohort and within analysis families defined for multiplicity control in the present report: HbO core metrics (mean, area under the curve [AUC], and slope across five regions; 15 tests), FC/network metrics (6 tests), and causal metrics (2 tests). *q* < 0.05 was considered significant; the correction is summarised by cohort and analysis family in Supplementary Table S8. The patient acupuncture protocol was registered on 15 October 2024. The public registry did not prospectively enumerate the detailed regional, network, causal, temporal, baseline-association, or machine-learning analysis families used in the present report, and these detailed analyses are therefore not described as prospectively preregistered. The healthy-participant cohort served as a parallel physiological comparator and was not part of the registered patient trial. Regional HbO contrasts were treated as the principal analysis in this report; baseline-adjusted and cohort-by-treatment models were treated as robustness analyses; and connectivity, network, causal, temporal, baseline-association, and machine-learning analyses were classified as exploratory (Supplementary Table S11).

For the baseline-state analysis, all 169 participants in the analysed cohort had paired Before and During recordings within the same session (Healthy–Real 60, Healthy–Sham 64, Patient–Real 22, and Patient–Sham 23). Baseline-versus-*Δ* correlations are reported as Spearman *ρ* with bootstrap 95% confidence intervals. Because correlating a baseline value with its subsequent change (Δ = During − Before) is biased toward a negative association by regression to the mean (mathematical coupling), we additionally examined the bias-free baseline-versus-During-level association as a sensitivity analysis. Analyses were performed in Python 3.11 (NumPy, SciPy.stats, statsmodels, scikit-learn) and SPSS 26.0.

## 3 Results

### 3.1 Baseline characteristics and signal quality

Baseline characteristics are shown in Table 1 and Supplementary Table S1. Patient CRS-R total scores were 8.27 ± 2.60 overall (MCS 10.16 ± 1.11; UWS 5.90 ± 1.86; Mann–Whitney *U*-test, *p* < 0.001). Etiologies were stroke (28/45, 62.2%), hypoxic–ischaemic encephalopathy (9/45, 20.0%), and traumatic brain injury (8/45, 17.8%). Mean time from injury to enrolment was 52.93 ± 14.12 weeks. Baseline characteristics did not differ between real and sham subgroups in either cohort (all *p* > 0.05).

**Table 1:** Demographic and clinical characteristics of pDOC patients (*n* = 45).

Characteristic	All patients (*n* = 45)	MCS (*n* = 25)	UWS (*n* = 20)	*p*
Age (years)	55.33 ± 16.18	56.12 ± 16.74	54.35 ± 15.83	0.740
Female, *n* (%)	23 (51.1)	13 (52.0)	10 (50.0)	1.000
CRS-R scores				
Total (0–23)	8.27 ± 2.60	10.16 ± 1.11	5.90 ± 1.86	<0.001***
Auditory (0–4)	1.51 ± 0.82	1.92 ± 0.57	1.00 ± 0.79	<0.001***
Visual (0–5)	1.31 ± 0.82	1.76 ± 0.66	0.75 ± 0.64	<0.001***
Motor (0–6)	1.53 ± 0.73	1.64 ± 0.76	1.40 ± 0.68	0.339
Oromotor (0–3)	1.40 ± 0.78	1.72 ± 0.68	1.00 ± 0.73	0.002**
Communication (0–2)	1.29 ± 0.82	1.72 ± 0.54	0.75 ± 0.79	<0.001***
Arousal (0–3)	1.22 ± 0.70	1.40 ± 0.65	1.00 ± 0.73	0.093
Clinical				
Time since injury (weeks)	52.93 ± 14.12	53.08 ± 13.77	52.75 ± 14.91	0.715
Stroke, *n* (%)	28 (62.2)	15 (60.0)	13 (65.0)	–
HIE, *n* (%)	9 (20.0)	6 (24.0)	3 (15.0)	–
TBI, *n* (%)	8 (17.8)	4 (16.0)	4 (20.0)	–
Real/sham allocation	22/23	13/12	9/11	–

Values are mean ± SD or *n* (%). *p*-values are from the Mann–Whitney *U*-test (continuous variables) or *χ*^2^/Fisher’s exact tests (categorical variables). *p < 0.05, **p < 0.01, and ***p < 0.001. HIE, hypoxic–ischaemic encephalopathy; TBI, traumatic brain injury.

Across the 507 phase-specific recordings, the overall mean SCI was 0.640, and 54.2% of the 35 measurement channels met the predefined SCI ≥ 0.75 criterion, corresponding to approximately 19 retained channels per recording. Recording-level summaries were descriptively similar in healthy participants and patients (mean SCI, 0.640 vs. 0.641; qualified-channel proportion, 53.4% vs. 56.4%; Supplementary Table S12). Because each participant contributed repeated Before, During, and After recordings, these values were not treated as statistically independent observations for inferential testing. The moderate retention reduced spatial sampling and is considered in the limitations.

### 3.2 Real acupuncture produces greater acute prefrontal HbO responses than superficial non-acupoint needling

**Figure 2:**
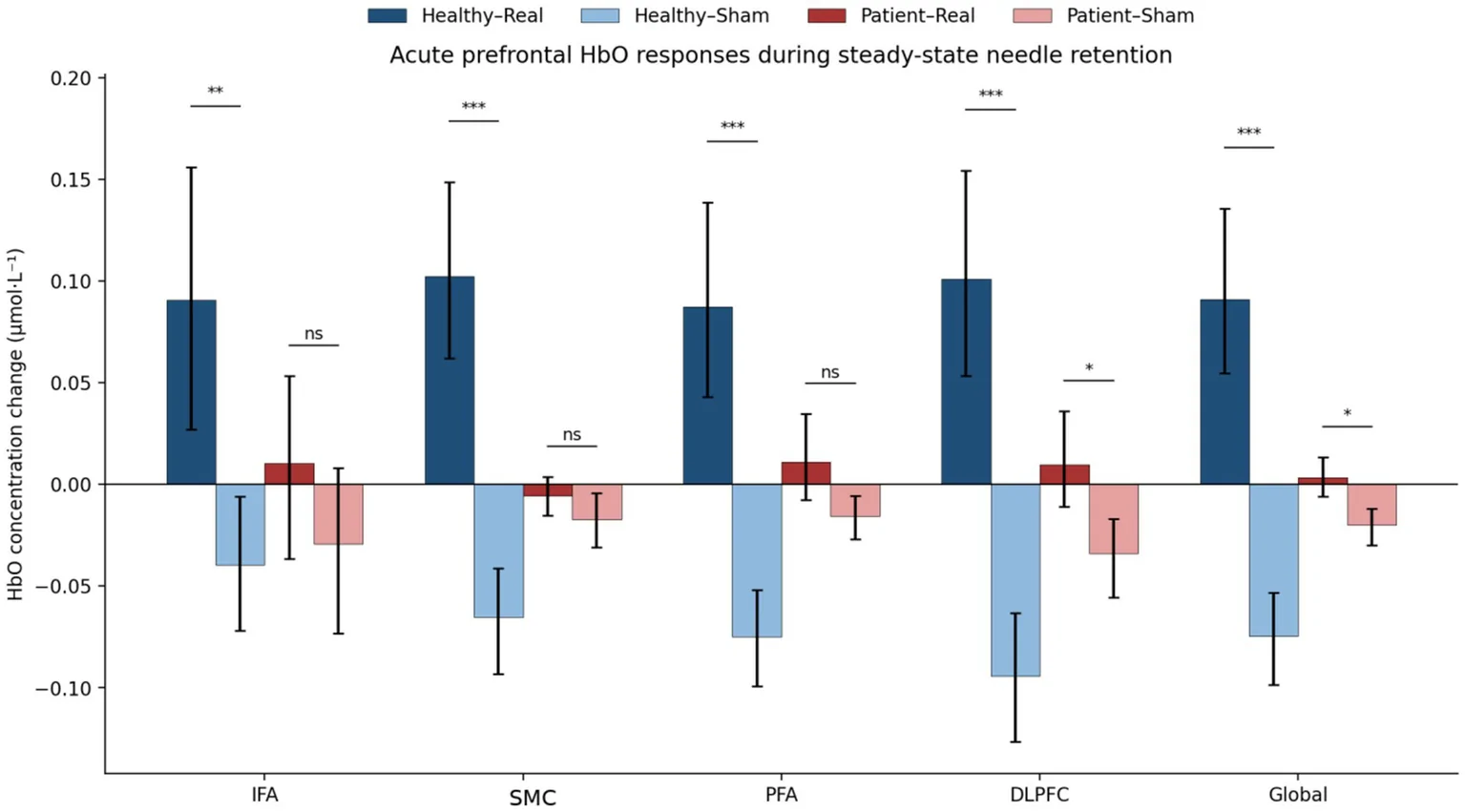
Acute prefrontal HbO responses during steady-state needle retention. Bars show group means and bootstrap 95% confidence intervals. Brackets compare real XNKQ acupuncture with superficial non-acupoint sham needling within each cohort. All five healthy contrasts survived FDR correction; in patients, DLPFC and Global survived correction, PFA was borderline, and IFA and SMC were not significant. *q < 0.05, **q < 0.01, and ***q < 0.001. HR, Healthy–Real; HS, Healthy–Sham; PR, Patient–Real; PS, Patient–Sham.

In healthy volunteers, real acupuncture produced greater During-phase HbO than sham across all four ROIs and the global prefrontal average: IFA *d* = 0.62, sensorimotor cortex (SMC) *d* = 1.12, PFA *d* = 1.07, DLPFC *d* = 1.14, and Global *d* = 1.20 (all *q* ≤ 0.0011). In pDOC patients, the response was smaller and regionally restricted. DLPFC (*d* = 0.80, *q* = 0.0397) and global HbO (*d* = 0.98, *q* = 0.0191) survived the 15-test HbO-family correction; PFA was borderline (*d* = 0.67, uncorrected *p* = 0.033, *q* = 0.0576), and SMC and IFA were not significant (*p* = 0.19–0.20). Thus, the patient response was not absent, but was less extensive than the healthy response (Table 2; Figure 2).

**Table 2:** Mean HbO concentration during the steady-state acupuncture phase, with real-versus-sham within-cohort effect sizes.

ROI	Healthy–Real (*n* = 60)	Healthy–Sham (*n* = 64)	Healthy *d* (95% CI)	Patient–Real (*n* = 22)	Patient–Sham (*n* = 23)	Patient *d* (95% CI)
IFA	0.091 ± 0.264	−0.039 ± 0.141	0.62**	0.010 ± 0.109	−0.029 ± 0.098	0.39 ns
SMC	0.103 ± 0.184	−0.065 ± 0.111	1.12***	−0.006 ± 0.025	−0.017 ± 0.034	0.39 ns
PFA	0.087 ± 0.192	−0.075 ± 0.098	1.07***	0.011 ± 0.049	−0.016 ± 0.028	0.67 ns
DLPFC	0.101 ± 0.209	−0.094 ± 0.126	1.14***	0.010 ± 0.058	−0.034 ± 0.050	0.80*
Global	0.091 ± 0.170	−0.075 ± 0.100	1.20***	0.003 ± 0.024	−0.020 ± 0.023	0.98*

HbO values are mean ± SD in μmol·L^−1^. Cohen’s *d* is real minus sham with bootstrap 95% confidence intervals from 2,000 resamples. *q < 0.05, **q < 0.01, and ***q < 0.001 after Benjamini–Hochberg correction within the 15-test HbO family for each cohort. Baseline-adjusted and interaction analyses are reported in Supplementary Table S7.

A four-group During-phase analysis of variance (ANOVA) was significant for all five HbO measures (Supplementary Table S5). Baseline-adjusted ANCOVA confirmed all five effects in healthy participants and PFA, DLPFC, and Global effects in patients. The cohort × treatment interaction was significant for SMC, PFA, DLPFC, and global HbO after FDR correction, but not for IFA, supporting a smaller treatment contrast in the pDOC cohort (Supplementary Table S7). Phase-wise HbO means for each ROI and group are shown in Supplementary Figure S1.

### 3.3 Exploratory FC-derived network summaries

**Figure 3:**
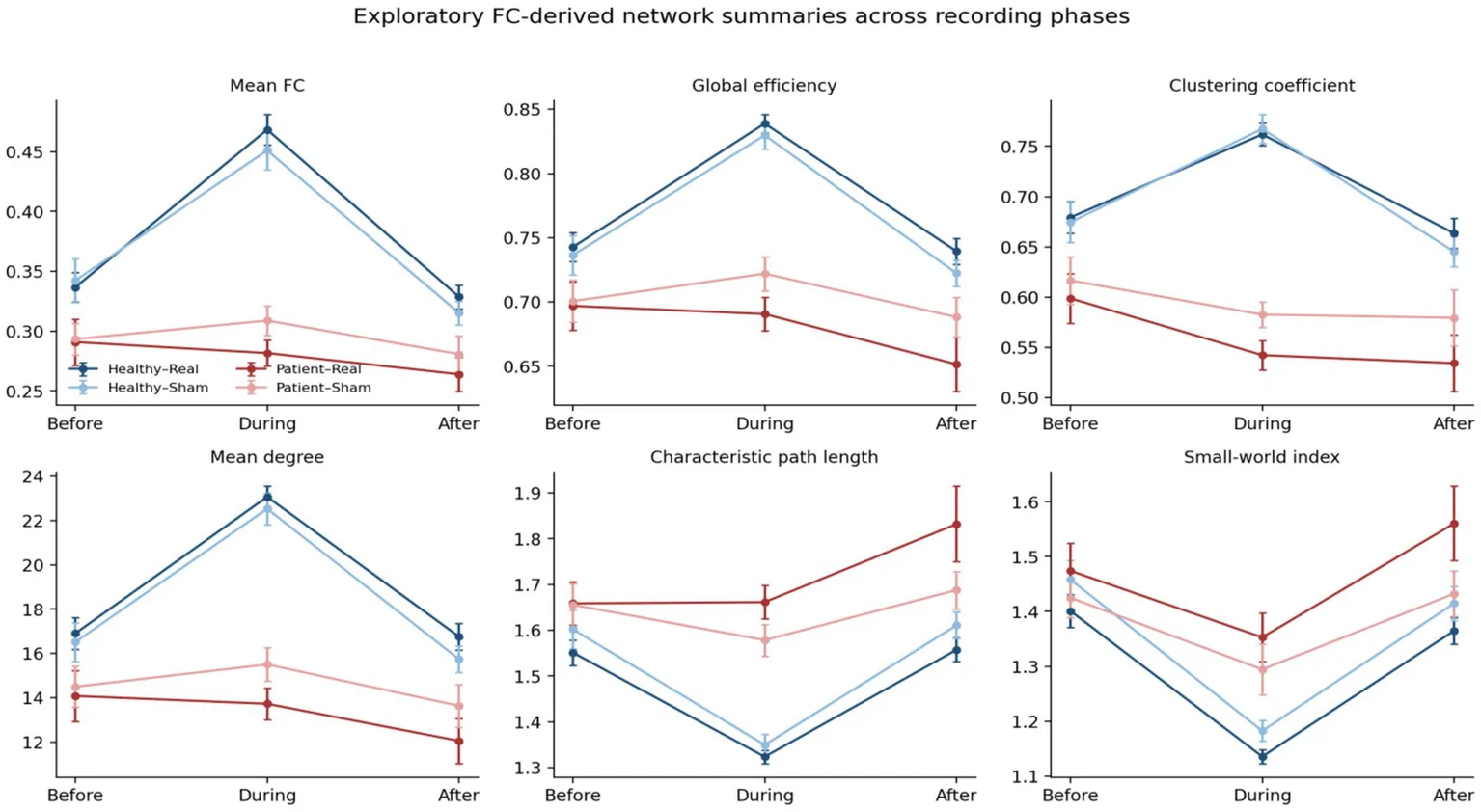
Exploratory FC-derived network summaries across Before, During, and After. Points show group means and error bars show SEM. Both real and sham stimulation were associated with phase-related changes, and no predefined real-versus-sham network contrast survived FDR correction. These summaries are descriptive and are not interpreted as confirmatory evidence of graph-theoretic network reorganisation.

In healthy volunteers, several integration-like summaries increased from Before to During under both real and sham stimulation (Figure 3; Supplementary Table S2). Because the real-versus-sham contrasts were small and did not survive FDR correction, these changes cannot be attributed specifically to the real XNKQ protocol.

**Figure 4:**
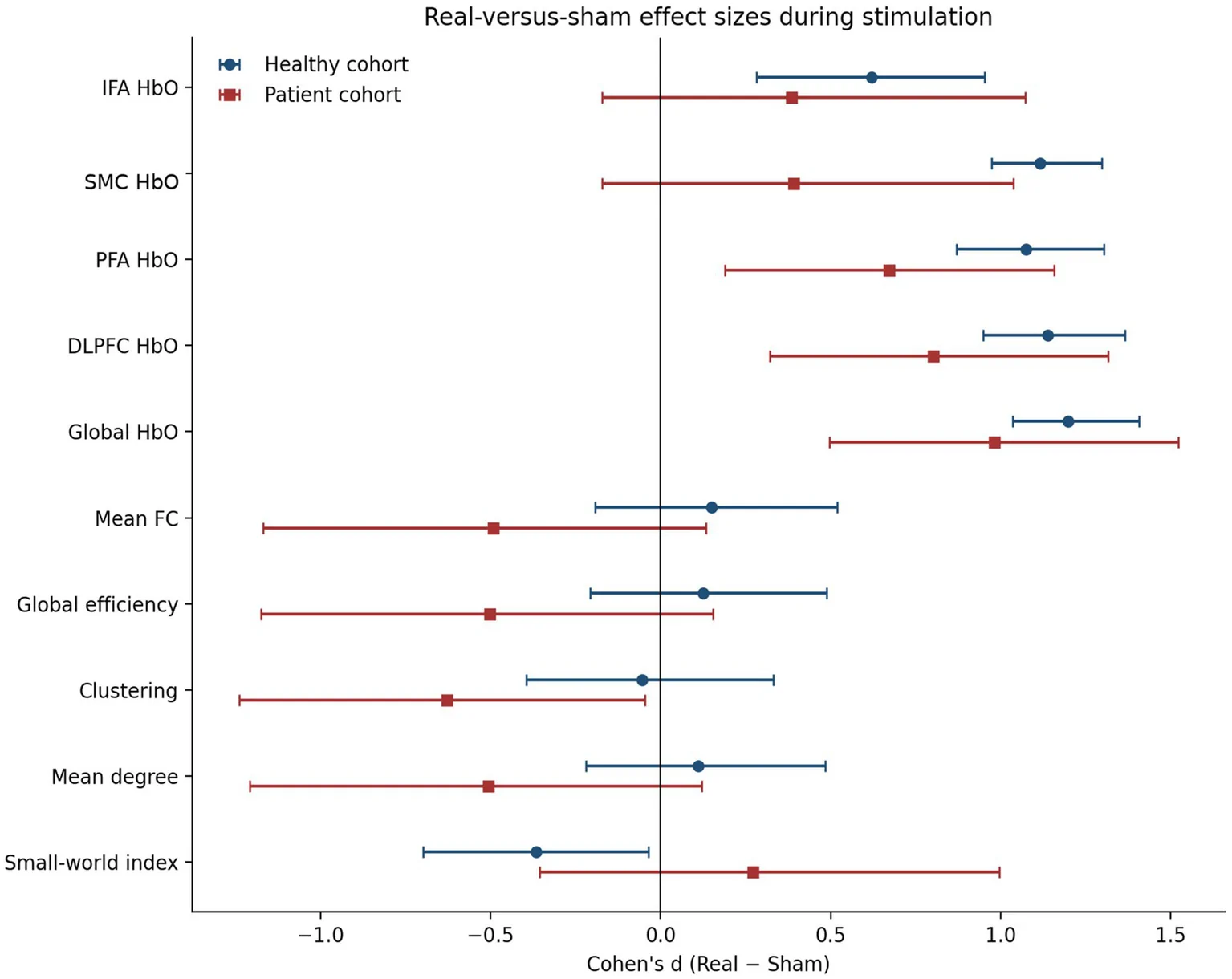
Real-versus-sham effect-size forest plot for the During phase. Cohen’s *d* is shown separately for healthy participants and pDOC patients with bootstrap 95% confidence intervals. HbO effects were broad in healthy participants and regionally restricted in patients. Network effects were heterogeneous and did not survive FDR correction.

In pDOC patients, several network summaries changed in the opposite descriptive direction, including lower mean FC, mean degree, clustering, and global efficiency under real than sham stimulation (Supplementary Table S3). However, none of the six predefined FC/network contrasts survived FDR correction in either cohort (Figure 4). The direction and biological meaning of these patterns are therefore uncertain, and the findings are retained only as hypothesis-generating summaries.

### 3.4 Baseline values and within-session network change: exploratory paired analysis

**Figure 5:**
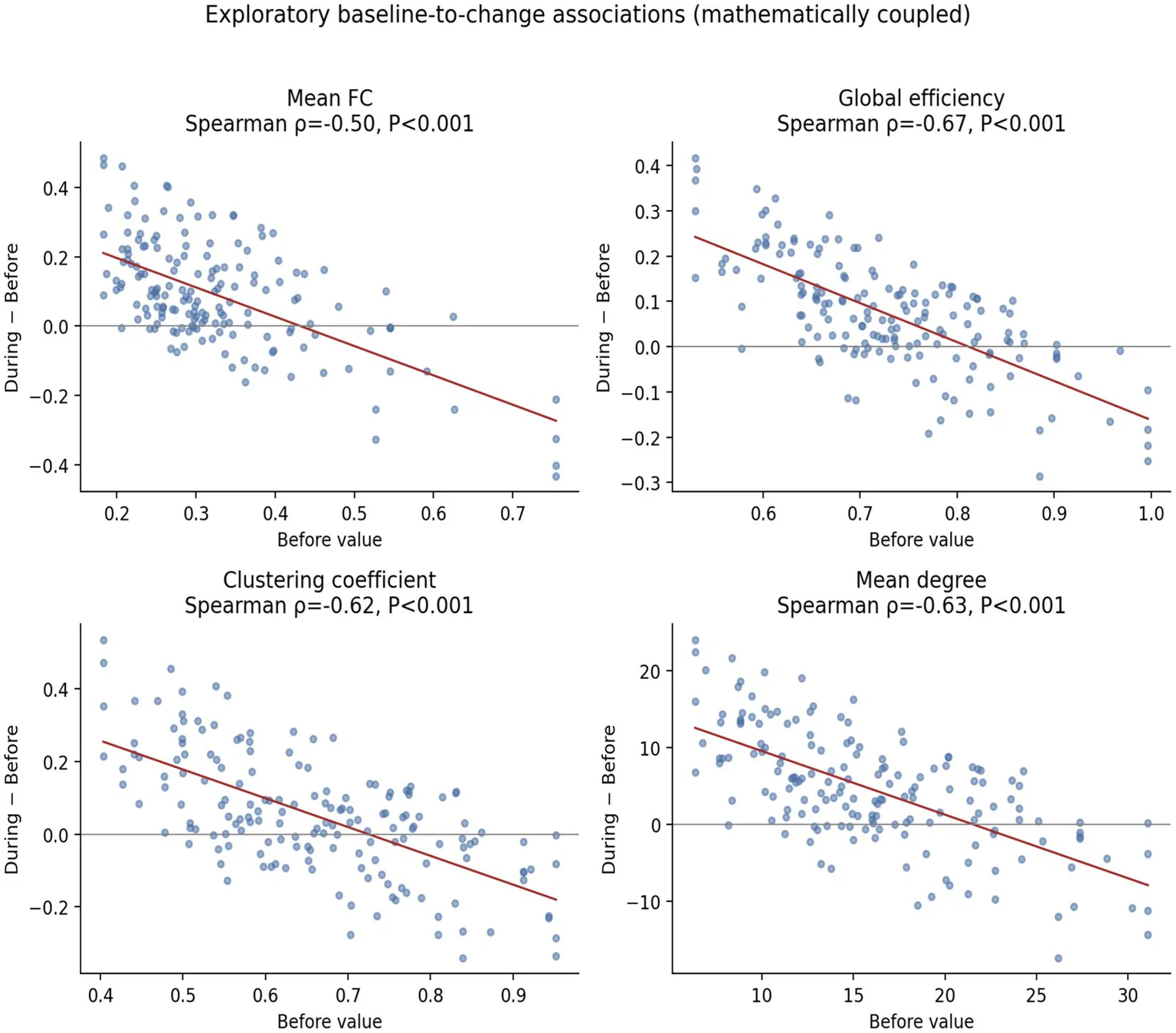
Exploratory baseline-to-change associations. Subject-level paired Before-and-During data are shown for four network summaries (*n* = 169). Because baseline is mathematically included in the change score, the negative slopes are expected to be inflated and are not interpreted as evidence that baseline state predicts within-session change.

Using paired Before and During recordings from 169 participants, baseline-versus-change correlations were negative: mean FC *ρ* = −0.50, global efficiency *ρ* = −0.67, clustering coefficient *ρ* = −0.62, mean degree *ρ* = −0.63, and local efficiency *ρ* = −0.67 (all *p* < 0.001; Table 3 and Figure 5).

**Table 3:** Exploratory baseline-to-change and baseline-to-during correlations.

Metric	Baseline versus Δ *ρ*	*p*	Baseline versus During ρ	*p*/*n*
Mean FC	−0.50	<0.001	+0.15	0.045/169
Global efficiency	−0.67	<0.001	+0.17	0.028/169
Clustering coefficient	−0.62	<0.001	+0.22	0.005/169
Mean degree	−0.63	<0.001	+0.17	0.025/169
Local efficiency	−0.67	<0.001	+0.19	0.012/169

All analyses used subject-level paired Before and During recordings (*n* = 169). Baseline-versus-change coefficients are mathematically coupled and susceptible to regression-to-the-mean inflation; baseline-versus-During correlations are shown as a less biased sensitivity analysis.

These coefficients are intrinsically biased toward negative values because the baseline term is included in Δ = During − Before. The bias-free baseline-versus-During correlations were only weakly positive (*ρ* = 0.15–0.22; *p* = 0.005–0.045), consistent with limited test–retest stability rather than a strong compensatory mechanism. The baseline-to-change analysis is therefore reported only as an exploratory, mathematically coupled observation and is not interpreted as predictive.

### 3.5 Temporal dynamics and exploratory machine-learning sensitivity analysis

Temporal analysis of the hemodynamic response (Supplementary Table S4) showed that real acupuncture in healthy participants produced an earlier hemodynamic peak (mean peak time 40.1–46.5% of the window) than sham (51.8–59.8%) and a more sustained plateau (recovery ratio 0.49–0.54 versus 0.63–0.77). Patients showed intermediate temporal profiles. These temporal differences are presented descriptively in Supplementary Table S4.

Participant-grouped internal validation provided only modest discrimination. Real-versus-sham classification was above chance in healthy participants but remained near chance in the pDOC cohort (best accuracy 53.3%, AUC 0.556; Supplementary Table S6). These results are presented only as exploratory sensitivity analyses and do not support individual-level clinical classification.

### 3.6 Exploratory directed-connectivity summaries

Group-level mean Granger-causality and transfer-entropy values are reported in Supplementary Table S9. Because these directed-connectivity analyses were exploratory and no pairwise inferential family was pre-specified, they are presented descriptively and are not used to support a directional network mechanism.

## 4 Discussion

The strongest finding is the acute HbO contrast between real XNKQ acupuncture and superficial non-acupoint needling. The effect was broad across the assessed prefrontal regions in healthy participants, but more restricted in pDOC patients, where DLPFC and global HbO were the most consistent FDR-corrected effects. This demonstrates an acute hemodynamic response and should not be read as evidence of clinical benefit, preserved consciousness, or recovery potential.

The FC-derived network summaries showed descriptively different phase-related patterns between cohorts, but no predefined network contrast survived FDR correction. Lower integration-like values in pDOC patients under real stimulation could reflect neural perturbation, altered vigilance, motion, systemic physiology, or signal-quality effects. Their direction and clinical meaning remain unknown.

The regional HbO pattern is compatible with the broader acupuncture neuroimaging literature (6, 7, 9, 33). The multisite XNKQ protocol provides facial and limb somatosensory input that may engage the distributed cortical and subcortical systems. Nevertheless, the present fNIRS measurements provide only a cortical hemodynamic readout and do not establish a specific afferent pathway. A prefrontal hemodynamic response must not be equated with awareness, conscious processing, or therapeutic engagement. The dual-cohort design suggests that the magnitude and regional extent of the acute HbO contrast differ between healthy and injured brains; the exploratory network observations require independent replication.

Our regional HbO results are consistent with the small but coherent fNIRS literature on acute cortical responses to acupuncture. Si et al. found that manual acupuncture with deqi modulated prefrontal and motor HbO and connectivity relative to a tactile control (18), and Cao et al. reported DLPFC and primary somatosensory activation under reinforcing-reducing manipulation (33). The broad prefrontal HbO increase we observed in healthy participants agrees with these reports and with the multisite somatosensory engagement described in the XNKQ protocol using fMRI (6). In pDOC, Wen et al. reported increased prefrontal HbO during acupuncture but without a sham comparator (8), and Liu et al. found DLPFC, M1, and S1 activation with connectivity changes after one real-versus-sham session (19). Our patient data agree with these studies in pointing to the DLPFC as the most consistently engaged region. What the present design adds is the superficial non-acupoint sham and the FDR control, which show that a measurable prefrontal HbO contrast survives a stricter analysis than earlier studies in this population applied.

Why was the response broad in healthy participants but confined to the DLPFC and the global average in patients? Several explanations are plausible and need not be mutually exclusive. Severe acquired brain injury disrupts thalamocortical and long-range cortical circuits that support integrated cortical responses (14), which can both weaken and spatially narrow a stimulus-evoked hemodynamic response. Cerebral blood flow regulation and neurovascular coupling are also commonly altered after acquired brain injury (34), which would further reduce the evoked signal. Lower signal-to-noise and heterogeneous structural damage in this cohort would affect smaller ROIs most, such as the two-channel IFA. The relative preservation of a DLPFC and global response supports the role of the DLPFC in frontoparietal systems linked to the recovery of consciousness, although our cortical measurements cannot identify the circuits involved. We therefore interpret the regional restriction as reduced and spatially narrower cortical reactivity in the injured brain, not as a marker of the depth of consciousness.

Our network summary should be read against the resting-state fNIRS literature in pDOC, which describes disrupted frontal connectivity and altered graph topology in minimally conscious and unresponsive patients (15–17). Some of that work reports connectivity features that separate levels of consciousness (17), and Liu et al. described session-related connectivity changes after acupuncture (19). In our data, by contrast, no real-versus-sham network contrast survived FDR correction in either cohort. Several methodological features may have reduced sensitivity. We regressed out the global component, grouped the analysis by participant, matched network density, and applied FDR correction within the predefined analysis families, while moderate channel retention and the physiologically active sham further limited sensitivity. This does not mean that no network effect exists. The lower integration-like values seen in patients under real stimulation are compatible with afferent input perturbing a vulnerable or already reorganised network, but their direction and clinical meaning cannot be established here. Because averaging across channels without short-separation measurement leaves the signal open to systemic and extracerebral influence (35), these exploratory network results in particular should be read with caution.

These findings should not be confused with the growing use of fNIRS to detect covert awareness in disorders of consciousness. Recent studies show that fNIRS can identify command-following and volitional brain activity at the bedside after acute severe brain injury (21–23). Those paradigms probe residual volitional cognition. The present study instead measures the acute, stimulus-driven cortical hemodynamic response to a passive intervention, so a prefrontal HbO increase here reflects evoked cortical reactivity rather than preserved awareness or command-following. In short, the convergent activation findings support the feasibility of bedside fNIRS for capturing acupuncture-related cortical responses in pDOC, whereas the null network results and the absence of longitudinal outcomes limit interpretation. The data show an acute, regionally organised hemodynamic signature that differs between healthy and injured brains, but not its mechanism or its link to clinical recovery.

The sham control is not physiologically inert. Superficial needling can produce somatosensory, expectancy, and autonomic effects. Accordingly, the present result is described as a greater response to the real XNKQ protocol than to superficial non-acupoint needling, but not as proof of strict acupoint specificity. Sham did not produce a comparable positive HbO pattern, but it was associated with phase-related network changes in healthy participants.

The baseline-to-change correlations are the weakest finding because of mathematical coupling. The weak positive baseline-versus-During associations do not support a strong compensatory mechanism. Although the responsiveness of the brain to external stimulation is known to depend on its state at the time of stimulation (36), the present design cannot separate any such state dependence from regression to the mean. Baseline fNIRS should not be presented as a predictor or clinical biomarker on the basis of these data.

### 4.1 Clinical implications

In the broader landscape of pDOC therapies, evidence remains limited, with the strongest support currently available for amantadine and select non-invasive neuromodulation approaches (37). Acupuncture is clinically used and produces measurable neurophysiological signatures, but its therapeutic efficacy remains unproven in the present study. Where our results can contribute is narrow but concrete. They show that a bedside, needle-compatible optical measurement can register an acute cortical response to acupuncture in patients who cannot report sensation, and that this response is larger for the real protocol than for superficial non-acupoint needling. Such an objective read-out could, in principle, help standardise stimulation and monitor cortical engagement in future trials. It does not by itself show that the response is therapeutic. A change in prefrontal HbO during a single session says nothing about whether repeated sessions improve CRS-R scores, diagnostic status or long-term recovery, and only a longitudinal, adequately powered trial with clinical end points can address that question.

### 4.2 Limitations

The patient sample (*n* = 45) is small for subgroup inference and machine-learning validation. fNIRS measures cortex only; thalamic and brainstem structures critical for consciousness are inaccessible (38). The IFA ROI has only two channels, limiting the reliability of intra-IFA connectivity estimates. Offline SCI pruning retained 54.2% of measurement channels, corresponding to approximately 19 of 35 channels per recording. No gross cohort-level quality imbalance was evident in the recording-level descriptive summaries, but the moderate spatial sampling may reduce precision, particularly for the two-channel IFA ROI and for channel-level connectivity and network summaries. The single-session design captures only acute, transient effects; longitudinal follow-up is needed to evaluate cumulative effects of repeated sessions. We did not quantify deqi or pain sensations; this was unavoidable in pDOC patients and limits comparison with acupuncture trials in fully communicative participants. A central methodological limitation is the absence of short-separation channels: although patients were continuously monitored with bedside vital-sign monitors and remained hemodynamically stable throughout the sessions, continuous heart-rate, blood-pressure, oxygen-saturation, and respiration traces were not stored for offline analysis; cortical HbO responses therefore cannot be fully separated from extracerebral and systemic contributions such as scalp blood flow and autonomic arousal, to which acupuncture is known to give rise. Consistent with this, HbR did not show the canonical decrease under real stimulation but changed in the same direction as HbO in the healthy cohort and in most patient regions, a pattern compatible with a blood volume-dominated response and with an extracerebral or systemic contribution that the present montage cannot exclude (35). Averaging HbO across channels within each ROI, without short-separation regression, can also yield a signal that partly reflects global systemic physiology or global cerebral activity rather than spatially localised cortical activation. This may reduce sensitivity to localised cerebral changes and limit the spatial specificity of the findings, and the concern applies to the regional HbO outcomes and not only to the connectivity analysis. This confound is particularly relevant to the connectivity-derived findings and is one reason they are reported as exploratory; prospective use of short-separation channels and continuous systemic monitoring is a priority for replication. Connectivity-derived network summaries were retained as exploratory and should not be overinterpreted as confirmatory fixed-threshold graph effects. The paired Before-and-During baseline-to-change analysis is subject to regression-to-the-mean inflation; the baseline-dependence result is therefore hypothesis-generating only. Further limitations concern reporting completeness: the CRS-R was administered by a single trained rater, so inter-rater reliability was not assessed; the healthy cohort was predominantly male and sex effects on the fNIRS measures were not tested, so residual influence of this imbalance cannot be excluded; and routine clinical medications were not prescribed as part of the study protocol or systematically modelled as covariates, so an influence of medication exposure on neurovascular coupling or cortical hemodynamic responses cannot be excluded. In summary, the study shows a broad acute prefrontal HbO contrast in healthy participants and a more restricted DLPFC/global contrast in pDOC patients; network observations were exploratory and did not survive FDR correction; and it cannot determine whether these acute signatures translate into recovery of consciousness or clinical benefit, which will require longitudinal, repeated-session designs with clinical follow-up. The exploratory machine-learning analysis used participant-grouped internal cross-validation but lacked an independent external test set and is not evidence of clinical utility.

## 5 Conclusion

Real XNKQ acupuncture was associated with greater acute prefrontal HbO responses than superficial non-acupoint needling. The response was broad in healthy participants and more restricted in pDOC patients, where DLPFC and global HbO were the most consistent effects. These data do not establish clinical benefit, recovery of consciousness, preserved awareness, or strict acupoint specificity. FC-derived network and baseline association results remain exploratory and require independent validation.

## Data Availability

The datasets presented in this article are not readily available because individual-level data from pDOC patients are subject to privacy and ethical restrictions. De-identified participant-level fNIRS feature data and the analysis code may be made available upon reasonable request, subject to approval by the Ethics Committee of Binzhou Medical University Hospital and a data-use agreement protecting patient privacy. Requests to access the datasets should be directed to the corresponding authors. Group-level summary data underlying the figures and supplementary tables are included in the article and Supplementary Material.
