# Supplementary material for: Acute prefrontal hemodynamic responses to Xingnao Kaiqiao acupuncture in healthy participants and patients with prolonged disorders of consciousness
Source: Front Neurol. 2026 Aug 28;17:1889514. doi: 10.3389/fneur.2026.1889514 (PMC13561800; doi:10.3389/fneur.2026.1889514)
Supplement: Supplementary file 1 [file Data_sheet_1.docx]

**Supplementary Materials**

Acute Prefrontal Hemodynamic Responses to Xingnao Kaiqiao Acupuncture in Healthy Participants and Patients with Prolonged Disorders of Consciousness

# Contents

Supplementary Methods

**Table S1. Demographic characteristics of healthy controls**

**Table S2. Exploratory FC-derived network summaries across all groups and phases.**

**Table S3. Functional-connectivity (Fisher-z) values during the acupuncture phase**

**Table S4. Temporal-dynamic features of the hemodynamic response**

**Table S5. Four-group one-way ANOVA for HbO mean across the four study groups**

**Table S6. Exploratory machine-learning classification performance under participant-grouped five-fold internal cross-validation.**

**Table S7. During-phase Real-versus-Sham HbO comparisons, baseline-adjusted ANCOVA and cohort × treatment interactions.**

**Table S8. Benjamini–Hochberg FDR-correction summary by cohort and analysis family.**

**Table S9. Granger causality and transfer entropy summary**

**Table S10. Region-level deoxyhemoglobin (HbR) responses during the steady-state acupuncture phase.**

**Table S11. Analysis hierarchy used in the present report.**

**Table S12. Recording-level descriptive signal-quality summaries by cohort.**

**Figure S1. Mean HbO across the three recording phases by ROI and group. Points show group means and error bars show SEM. The healthy cohort showed a broad positive Real-versus-Sham contrast during stimulation, whereas the patient contrast was smaller and regionally restricted.**

**Figure S2. Graph-theoretic concepts illustrated on toy networks**

# Supplementary Methods

### fNIRS signal pre-processing pipeline

Raw optical-intensity signals at 730 and 850 nm were converted to optical density. Motion artefacts were identified and corrected on the two-wavelength optical-density signals before the final hemodynamic filter. After SCI-based channel pruning, qualified optical-density signals were converted to relative HbO and HbR concentration changes using the modified Beer–Lambert law with age-adjusted differential pathlength factors and then filtered with a zero-phase third-order Butterworth band-pass filter (0.01–0.15 Hz).

### Motion correction and SCI-based channel-quality assessment

Motion artefacts were detected using a moving standard-deviation criterion and repaired with spline interpolation and temporal derivative distribution repair. After motion correction and before the final 0.01–0.15 Hz hemodynamic filter, the scalp coupling index was calculated from the motion-corrected two-wavelength optical-density signals after isolation of the cardiac band. Channels with SCI < 0.75 were excluded from subsequent analyses. Across the 507 recordings, the overall mean SCI was 0.640, and 54.2% of the 35 measurement channels met the SCI ≥ 0.75 criterion, corresponding to approximately 19 retained channels per recording. Recording-level descriptive values were similar in the healthy and patient cohorts (Table S12). Because each participant contributed three repeated phase recordings, no independent-record inferential comparison was used. ROI summaries were averaged across retained channels. The principal HbO dataset (the four ROI-averaged values and the global channel-average) contained no missing outcome values, and no across-participant imputation was used. The moderate absolute retention reduced spatial sampling and is acknowledged as a limitation.

We applied a standard-deviation-based motion-detection algorithm with a sliding 1-s window. Time-points whose SD exceeded 5× the baseline SD were flagged as motion artefacts and corrected via spline interpolation (order = 5) combined with temporal derivative distribution repair (TDDR) (Scholkmann et al., 2010; Fishburn et al., 2019). Across the 507 recordings (after excluding 3 healthy participants with incomplete three-phase fNIRS data), an average of 2.3% of samples were flagged per channel (range 0–11.7%); these proportions were retained as descriptive quality information and were not used as an additional channel-exclusion criterion in the final analysis.

### Exploratory FC-derived network analysis

Functional-connectivity matrices were summarised using FC-derived network features, including degree-like connectivity strength, clustering coefficient, characteristic path length, global efficiency, local efficiency and small-world index. Density-matched network representations were examined descriptively to reduce dependence on overall connection strength. Because graph summaries are threshold-dependent and threshold robustness was not treated as established, these variables are retained only as exploratory descriptors and are not interpreted as confirmatory graph-topology endpoints.

### Granger causality and transfer entropy

ROI-level time-series were obtained by averaging HbO across constituent channels. Pairwise Granger causality was computed using a vector autoregressive (VAR) model with order selected by Bayesian information criterion (range 5–9 across recordings). Transfer entropy was estimated using the JIDT toolbox with k-nearest-neighbour entropy estimation (k = 4). Both metrics were computed for all 12 directed ROI pairs.

Machine-learning feature construction and validation

The exploratory machine-learning analysis used random forest, linear support-vector machine and logistic regression to span complementary model families. Cross-validation was grouped by anonymized participant identifier, with all three phase recordings from the same participant assigned to the same fold. Imputation, standardisation when required and univariate feature selection retaining the top 50 features were fitted only within each training fold and then applied to held-out data. Detailed reporting was restricted to all-phase tasks; During-only sensitivity classifications and the previous main-text machine-learning table and figure were removed to reduce overinterpretation. The analysis was internally validated only and was not used to support diagnostic or clinical utility.

# Supplementary Tables

**Table S1. Demographic characteristics of healthy controls (*n* = 127 enrolled; n = 124 analysed after excluding 3 with incomplete fNIRS).**

| **Characteristic** | **All healthy (enrolled n=127; analysed n=124)** | **Real (n=63 enrolled; n=60 analysed)** | **Sham (n=64)** | **P** |
| --- | --- | --- | --- | --- |
| Age (years) | 56.33 ± 10.26 | 55.89 ± 10.54 | 56.77 ± 9.98 | 0.632 |
| Female, n (%) | 25 (19.7) | 12 (19.0) | 13 (20.3) | 0.858 |
| Body-mass index (kg·m⁻²) | 23.4 ± 2.8 | 23.5 ± 2.7 | 23.3 ± 2.9 | 0.671 |
| Right-handed, n (%) | 122 (96.1) | 61 (96.8) | 61 (95.3) | 0.999 |
| Years of education | 13.8 ± 3.2 | 13.7 ± 3.0 | 13.9 ± 3.3 | 0.733 |

Note: Data are mean ± SD or n (%). No significant differences between real and sham subgroups in any baseline characteristic. Independent *t*-test for continuous variables, χ² test for categorical variables.

**Table S2. Exploratory FC-derived network summaries across all groups and phases.**

| **Group** | **Before** | **During** | **After** |
| --- | --- | --- | --- |
| Mean FC | | | |
| Healthy–Real | 0.3367 | 0.4684 | 0.3287 |
| Healthy–Sham | 0.3423 | 0.4511 | 0.3152 |
| Patient–Real | 0.2909 | 0.2817 | 0.2640 |
| Patient–Sham | 0.2934 | 0.3089 | 0.2808 |
| Mean degree | | | |
| Healthy–Real | 16.90 | 23.07 | 16.76 |
| Healthy–Sham | 16.51 | 22.53 | 15.74 |
| Patient–Real | 14.08 | 13.73 | 12.05 |
| Patient–Sham | 14.50 | 15.50 | 13.64 |
| Clustering coefficient | | | |
| Healthy–Real | 0.6793 | 0.7618 | 0.6635 |
| Healthy–Sham | 0.6745 | 0.7672 | 0.6452 |
| Patient–Real | 0.5988 | 0.5424 | 0.5344 |
| Patient–Sham | 0.6166 | 0.5827 | 0.5795 |
| Characteristic path length | | | |
| Healthy–Real | 1.5513 | 1.3236 | 1.5573 |
| Healthy–Sham | 1.6022 | 1.3496 | 1.6110 |
| Patient–Real | 1.6588 | 1.6616 | 1.8321 |
| Patient–Sham | 1.6556 | 1.5785 | 1.6880 |
| Global efficiency | | | |
| Healthy–Real | 0.7427 | 0.8389 | 0.7394 |
| Healthy–Sham | 0.7364 | 0.8298 | 0.7223 |
| Patient–Real | 0.6969 | 0.6906 | 0.6514 |
| Patient–Sham | 0.7006 | 0.7220 | 0.6882 |
| Local efficiency | Local efficiency | Local efficiency | Local efficiency |
| Healthy–Real | 0.8393 | 0.8782 | 0.8329 |
| Healthy–Sham | 0.8234 | 0.8837 | 0.8132 |
| Patient–Real | 0.7950 | 0.7799 | 0.7625 |
| Patient–Sham | 0.8081 | 0.7910 | 0.7887 |
| Small-world index | Small-world index | Small-world index | Small-world index |
| Healthy–Real | 1.4010 | 1.1356 | 1.3649 |
| Healthy–Sham | 1.4583 | 1.1823 | 1.4148 |
| Patient–Real | 1.4742 | 1.3529 | 1.5605 |
| Patient–Sham | 1.4252 | 1.2944 | 1.4325 |

Note: Values are group means of connectivity-derived network summaries from the analysis feature table. These variables are retained as exploratory descriptors and are not interpreted as confirmatory fixed-threshold graph endpoints. Bold values mark the During phase, which is the primary stimulation window.

**Table S3. Functional-connectivity (Fisher-z) values during the acupuncture phase.**

| **FC metric** | **Healthy–Real** | **Healthy–Sham** | **Patient–Real** | **Patient–Sham** |
| --- | --- | --- | --- | --- |
| Whole-brain FC | 0.468 | 0.451 | 0.282 | 0.309 |
| IFA–IFA | 0.667 | 0.687 | 0.472 | 0.575 |
| SMC–SMC | 0.318 | 0.357 | 0.263 | 0.289 |
| PFA–PFA | 0.461 | 0.433 | 0.293 | 0.304 |
| DLPFC–DLPFC | 0.471 | 0.477 | 0.302 | 0.300 |
| IFA–SMC | 0.442 | 0.428 | 0.263 | 0.279 |
| IFA–PFA | 0.440 | 0.433 | 0.259 | 0.289 |
| IFA–DLPFC | 0.357 | 0.357 | 0.272 | 0.272 |
| SMC–PFA | 0.464 | 0.448 | 0.284 | 0.310 |
| SMC–DLPFC | 0.470 | 0.458 | 0.272 | 0.300 |
| PFA–DLPFC | 0.470 | 0.456 | 0.292 | 0.312 |

*Note: Fisher-z-transformed Pearson correlation coefficients averaged across all channel pairs within (intra) or between (inter) each ROI. Group means during the 8-min During-phase window.*

**Table S4. Temporal-dynamic features of the hemodynamic response during acupuncture.**

| **Metric** | **Healthy–Real** | **Healthy–Sham** | **Patient–Real** | **Patient–Sham** |
| --- | --- | --- | --- | --- |
| IFA | | | | |
| Peak time (% of window) | 43.5 ± 28.4 | 59.8 ± 24.9 | 48.4 ± 24.7 | 52.2 ± 28.2 |
| Recovery ratio | 0.54 ± 0.38 | 0.77 ± 0.25 | 0.73 ± 0.27 | 0.76 ± 0.28 |
| SMC | | | | |
| Peak time (% of window) | 41.6 ± 23.0 | 57.2 ± 23.0 | 57.3 ± 29.8 | 55.0 ± 20.7 |
| Recovery ratio | 0.53 ± 0.37 | 0.68 ± 0.31 | 0.70 ± 0.35 | 0.76 ± 0.26 |
| PFA | | | | |
| Peak time (% of window) | 46.5 ± 23.9 | 51.8 ± 26.9 | 55.2 ± 25.6 | 50.0 ± 23.4 |
| Recovery ratio | 0.52 ± 0.35 | 0.63 ± 0.32 | 0.64 ± 0.35 | 0.68 ± 0.33 |
| DLPFC | | | | |
| Peak time (% of window) | 40.1 ± 31.4 | 56.3 ± 26.7 | 54.7 ± 25.9 | 56.8 ± 27.6 |
| Recovery ratio | 0.49 ± 0.40 | 0.63 ± 0.35 | 0.73 ± 0.27 | 0.74 ± 0.30 |

*Note: Peak time: percentage of the 8-min window at which HbO reached its maximum. Recovery ratio: mean HbO in the last 2 min divided by peak HbO. Lower recovery ratio in real-acupuncture conditions indicates a more sustained plateau response. Values are mean ± SD.*

**Table S5. Four-group one-way ANOVA for HbO mean across the four study groups (Healthy–Real, Healthy–Sham, Patient–Real, Patient–Sham).**

| **ROI** | **Phase** | **F statistic** | **P value** | **Significance** |
| --- | --- | --- | --- | --- |
| IFA | | | | |
| IFA | Before | 3.161 | 0.026 | * |
| IFA | During | 5.478 | 0.001 | ** |
| IFA | After | 3.537 | 0.016 | * |
| SMC | | | | |
| SMC | Before | 3.219 | 0.024 | * |
| SMC | During | 17.643 | <0.001 | *** |
| SMC | After | 0.480 | 0.697 | ns |
| PFA | | | | |
| PFA | Before | 3.870 | 0.010 | * |
| PFA | During | 15.913 | <0.001 | *** |
| PFA | After | 1.092 | 0.354 | ns |
| DLPFC | | | | |
| DLPFC | Before | 4.070 | 0.008 | ** |
| DLPFC | During | 17.841 | <0.001 | *** |
| DLPFC | After | 0.699 | 0.554 | ns |
| Global | | | | |
| Global | Before | 4.157 | 0.007 | ** |
| Global | During | 20.144 | <0.001 | *** |
| Global | After | 0.918 | 0.434 | ns |

Note: Four-group one-way ANOVA (df = 3, 165) testing whether the HbO mean differs across the four study groups at each phase. Significant Before-phase differences reflect pre-existing differences between the healthy and patient cohorts rather than a real-vs-sham imbalance; the within-cohort real-vs-sham subgroups were balanced at baseline (Table 1; Supplementary Table S7). * P < 0.05, ** P < 0.01, *** P < 0.001 after Benjamini–Hochberg FDR correction. ns, not significant. The within-cohort (Real-vs-Sham) one-way ANOVA F values referenced in the main text are reported separately in Table S7.

**Table S6. Exploratory machine-learning classification performance under participant-grouped five-fold internal cross-validation.**

| Task | RF | Linear SVM | Logistic | Best model | Accuracy | AUC |
| --- | --- | --- | --- | --- | --- | --- |
| Healthy vs Patient (all phases) | 78.11% | 71.79% | 70.41% | RF | 78.11% | 0.813 |
| Real vs Sham, healthy (all phases) | 65.32% | 59.68% | 58.87% | RF | 65.32% | 0.708 |
| Real vs Sham, patient (all phases) | 53.33% | 41.48% | 41.48% | RF | 53.33% | 0.556 |
| Real vs Sham, combined (all phases) | 63.31% | 61.93% | 62.72% | RF | 63.31% | 0.697 |
| Phase Before/During/After, healthy (3-class) | 59.14% | 59.95% | 58.60% | Linear SVM | 59.95% | — |
| Phase Before/During/After, patient (3-class) | 50.37% | 45.93% | 44.44% | RF | 50.37% | — |

Note: Estimates are from five-fold internal cross-validation grouped by anonymized participant identifier; all recordings from the same participant remained in the same fold. Imputation, scaling when required and feature selection were performed within each training fold. RF, random forest; Linear SVM, linear support-vector machine. AUC is reported only for binary tasks. These exploratory internal-validation results were not externally validated and should not be interpreted as diagnostic or clinical models.

**Table S7. During-phase Real-versus-Sham HbO comparisons, baseline-adjusted ANCOVA and cohort × treatment interactions.**

| **Analysis** | **ROI** | **Estimate / statistic** | **P and FDR q** | **Interpretation** |
| --- | --- | --- | --- | --- |
| Healthy During | IFA | Welch t = 3.39 | P = 0.0010; q = 0.0011 | Real > Sham |
| Healthy During | SMC | Welch t = 6.11 | P < 0.001; q < 0.001 | Real > Sham |
| Healthy During | PFA | Welch t = 5.87 | P < 0.001; q < 0.001 | Real > Sham |
| Healthy During | DLPFC | Welch t = 6.23 | P < 0.001; q < 0.001 | Real > Sham |
| Healthy During | Global | Welch t = 6.57 | P < 0.001; q < 0.001 | Real > Sham |
| Patient During | IFA | Welch t = 1.29 | P = 0.204; q = 0.220 | Not significant |
| Patient During | SMC | Welch t = 1.32 | P = 0.193; q = 0.220 | Not significant |
| Patient During | PFA | Welch t = 2.23 | P = 0.033; q = 0.0576 | Borderline |
| Patient During | DLPFC | Welch t = 2.68 | P = 0.0106; q = 0.0397 | Real > Sham |
| Patient During | Global | Welch t = 3.29 | P = 0.0020; q = 0.0191 | Real > Sham |
| ANCOVA Healthy | IFA | β = 0.135 | q = 0.0028 | Adjusted Real > Sham |
| ANCOVA Healthy | SMC | β = 0.168 | q < 0.001 | Adjusted Real > Sham |
| ANCOVA Healthy | PFA | β = 0.164 | q < 0.001 | Adjusted Real > Sham |
| ANCOVA Healthy | DLPFC | β = 0.195 | q < 0.001 | Adjusted Real > Sham |
| ANCOVA Healthy | Global | β = 0.167 | q < 0.001 | Adjusted Real > Sham |
| ANCOVA Patient | IFA | β = 0.040 | q = 0.217 | Not significant |
| ANCOVA Patient | SMC | β = 0.012 | q = 0.217 | Not significant |
| ANCOVA Patient | PFA | β = 0.027 | q = 0.0383 | Adjusted Real > Sham |
| ANCOVA Patient | DLPFC | β = 0.041 | q = 0.0192 | Adjusted Real > Sham |
| ANCOVA Patient | Global | β = 0.023 | q = 0.0032 | Adjusted Real > Sham |
| Cohort × treatment | IFA | β = −0.094 | q = 0.075 | Not significant |
| Cohort × treatment | SMC | β = −0.156 | q < 0.001 | Smaller effect in patients |
| Cohort × treatment | PFA | β = −0.137 | q < 0.001 | Smaller effect in patients |
| Cohort × treatment | DLPFC | β = −0.151 | q < 0.001 | Smaller effect in patients |
| Cohort × treatment | Global | β = −0.143 | q < 0.001 | Smaller effect in patients |

Note: Direct During-phase comparisons use Welch independent-samples t-tests. ANCOVA models adjust the During value for the corresponding Before value. Cohort × treatment models formally compare the adjusted treatment contrast between healthy participants and pDOC patients. FDR q values are reported within the stated analysis family.

**Table S8. Benjamini–Hochberg FDR-correction summary by cohort and analysis family.**

| **Analysis family** | **Cohort** | **N tests** | **Significant raw** | **Significant after FDR** |
| --- | --- | --- | --- | --- |
| HbO core (mean, AUC, slope × 5) | Healthy | 15 | 15 | 15 |
| HbO core (mean, AUC, slope × 5) | Patient | 15 | 9 | 6 |
| FC/network | Healthy | 6 | 1 | 0 |
| FC/network | Patient | 6 | 1 | 0 |

*Note: Tests organised by the analysis families defined for the revised report to reduce multiple-comparison burden. All within-family P values were sorted ascending and the largest passing rank (i × q / N ≥ P_i) was used as the cutoff (q = 0.05).*

**Table S9. Granger causality and transfer entropy summary across groups (During phase).**

| **Metric** | **Healthy–Real** | **Healthy–Sham** | **Patient–Real** | **Patient–Sham** |
| --- | --- | --- | --- | --- |
| Mean Granger causality | 13.782 | 10.649 | 5.062 | 4.788 |
| Mean transfer entropy | 0.377 | 0.406 | 0.536 | 0.478 |

Note: Values are group means during the 8-min During window. Granger causality was estimated from BIC-selected VAR models and transfer entropy with k-nearest-neighbour estimation. These directed-connectivity summaries are exploratory.

**Table S10. Region-level deoxyhemoglobin (HbR) responses during the steady-state acupuncture phase.**

| **ROI** | **Healthy–Real** | **Healthy–Sham** | **Patient–Real** | **Patient–Sham** | **HbO (H–Real, ref.)** |
| --- | --- | --- | --- | --- | --- |
| IFA | +0.009 | -0.066 | +0.001 | -0.043 | +0.091 |
| SMC | +0.050 | -0.028 | +0.001 | -0.021 | +0.103 |
| PFA | +0.023 | -0.073 | -0.005 | -0.028 | +0.088 |
| DLPFC | +0.041 | -0.048 | +0.036 | -0.016 | +0.101 |
| Global | +0.046 | -0.067 | +0.016 | -0.016 | +0.091 |

Values are mean HbR change (μmol·L⁻¹) during the steady-state retention window, referenced to the mean of the first 20 s of that recording window. HbO and HbR were derived from the two-wavelength optical density using the modified Beer–Lambert law (Prahl/Gratzer extinction coefficients; age-adjusted differential pathlength factors), with an increase in either chromophore expressed as a positive value. Under real acupuncture HbR therefore increased rather than showing the canonical decrease in the healthy cohort and in most patient regions, in the same direction as HbO. In the absence of short-separation channels this pattern is consistent with a blood-volume–dominated response with a possible extracerebral or systemic contribution; the HbR results are accordingly interpreted descriptively only and are not used as independent evidence for a specific neurovascular mechanism. The final column reproduces the Healthy–Real HbO means (Table 2) for comparison.

**Table S11. Analysis hierarchy used in the present report.**

| **Analysis** | **Status in present report** | **Rationale** |
| --- | --- | --- |
| Regional and global HbO Real-vs-Sham contrasts | Principal analysis | Directly addresses the primary mechanistic question; 15-test FDR family within each cohort. |
| Baseline-adjusted HbO and cohort × treatment interactions | Robustness analyses | Assess sensitivity to baseline values and formally compare treatment contrasts between cohorts. |
| Functional connectivity and FC-derived network summaries | Exploratory | No pre-defined network contrast survived FDR correction. |
| Granger causality / transfer entropy | Exploratory | Secondary directed-connectivity summaries. |
| Temporal dynamics and baseline associations | Exploratory | Baseline-to-change analysis is mathematically coupled. |
| Machine-learning classification | Exploratory internal validation | Participant-grouped five-fold cross-validation; no external test set. |

The patient acupuncture protocol was registered at the Chinese Clinical Trial Registry on 15 October 2024 (ChiCTR2400090915). The healthy-participant cohort was included as a parallel physiological comparator and was not part of the registered patient trial. This table describes the analysis hierarchy used in the present report and does not imply that every detailed exploratory analysis was specified in the public registry.

**Table S12. Recording-level descriptive signal-quality summaries by cohort.**

| **Metric** | **Healthy (372 recordings)** | **Patient (135 recordings)** | **Absolute difference** | **Analysis level** | **Interpretation** |
| --- | --- | --- | --- | --- | --- |
| Mean SCI | 0.640 | 0.641 | 0.001 | Recording-level descriptive | Similar |
| Qualified channels (%) | 53.4 | 56.4 | 3.0 percentage points | Recording-level descriptive | Similar |
| SCI criterion | ≥0.75 | ≥0.75 | — | Channel-wise | Applied to retention |

SCI was computed from the motion-corrected two-wavelength optical-density signals in the cardiac band and before the final hemodynamic filter. Channels with SCI < 0.75 were excluded. Across all 507 recordings, the mean SCI was 0.640 and 54.2% of measurement channels met the criterion, corresponding to approximately 19 retained channels per recording. The healthy and patient recording-level summaries were similar descriptively. Because each participant contributed repeated Before, During and After recordings, the table does not treat the 507 recordings as statistically independent observations. The moderate proportion of retained channels and absence of short-separation measurements remain limitations. The principal HbO dataset (the four ROI-averaged values and the global channel-average) contained no missing outcome values, and no across-participant imputation was used.

# Supplementary Figures

**Figure S1. Mean HbO across the three recording phases by ROI and group. Points show group means and error bars show SEM. The healthy cohort showed a broad positive Real-versus-Sham contrast during stimulation, whereas the patient contrast was smaller and regionally restricted.**

**Figure S2. Graph-theoretic concepts illustrated on toy networks. (A–C) Three reference topologies on 16 nodes with the same number of edges: a regular ring lattice has high clustering and long path length, a random graph has lower clustering and shorter path length, and a small-world graph combines relatively high clustering with relatively short path length. This figure is conceptual and is retained only to clarify the meaning of exploratory network-summary terms.**
